# Multi-mW, few-cycle mid-infrared continuum spanning from 500 to 2250 cm^−1^

**DOI:** 10.1038/lsa.2017.180

**Published:** 2018-02-23

**Authors:** Jinwei Zhang, Ka Fai Mak, Nathalie Nagl, Marcus Seidel, Dominik Bauer, Dirk Sutter, Vladimir Pervak, Ferenc Krausz, Oleg Pronin

**Affiliations:** 1 Max-Planck-Institute of Quantum Optics, Hans-Kopfermann-Str. 1, Garching 85748, Germany; 2 Ludwig-Maximilians-University Munich, Am Coulombwall 1, Garching 85748, Germany; 3 TRUMPF Laser GmbH, Aichhalder Straße 39, Schramberg D-78713, Germany

## Abstract

The demand for and usage of broadband coherent mid-infrared sources, such as those provided by synchrotron facilities, are growing. Since most organic molecules exhibit characteristic vibrational modes in the wavelength range between 500 and 4000 cm^−1^, such broadband coherent sources enable micro- or even nano-spectroscopic applications at or below the diffraction limit with a high signal-to-noise ratio^1, 2, 3^. These techniques have been applied in diverse fields ranging from life sciences, material analysis, and time-resolved spectroscopy. Here we demonstrate a broadband, coherent and intrinsically carrier-envelope-phase-stable source with a spectrum spanning from 500 to 2250 cm^−1^ (−30 dB) at an average power of 24 mW and a repetition rate of 77 MHz. This performance is enabled by the first mode-locked thin-disk oscillator operating at 2 μm wavelength, providing a tenfold increase in average power over femtosecond oscillators previously demonstrated in this wavelength range^4^. Multi-octave spectral coverage from this compact and power-scalable system opens up a range of time- and frequency-domain spectroscopic applications.

Octave-spanning and spectrally coherent radiation has enabled key advances in metrology and spectroscopic applications in the near infrared^5, 6^. Recent efforts by the research community seek to extend radiation with such characteristics to the mid-infrared (MIR)^7, 8, 9, 10^, especially to the range of 500–4000 cm^−1^ (2.5–20 μm), where organic molecules have fundamental vibrational absorption, allowing their unique identification^9, 11^. A source that can simultaneously cover the entire window holds promise for better specificity for biomedical applications^12^. In addition, a coherent spectrum with a Fourier limit equivalent to a few-cycle or even a single-cycle MIR pulse in the time domain^1, 8, 10^ will further benefit time-resolved pump-probe measurements^13^ and allow improved control of molecular excitation dynamics^14^. Moreover, when combined with carrier-envelope phase (CEP) stabilization and a 10 fs-scale sampling pulse, which can be routinely provided, the source can facilitate the use of electro-optical sampling (EOS)^15^ detection as a highly attractive alternative to Fourier transform spectroscopy with liquid-nitrogen cooled HgCdTe detectors.

Only a few experimental materials can lase in the MIR^16^. While quantum cascade lasers^17^, fiber supercontinuum sources^7^ and synchrotron infrared facilities^3, 18^ provide high power, broad bandwidth and diffraction-limited beams, or even a combination of all three, they lack the full spectral phase coherence critical for many of the aforementioned applications. Octave-spanning sources based on gas filaments can provide sub-cycle pulses^19^, but they require high pulse energy and careful handling to stabilize the output intensity.

Consequently, coherent coverage of the MIR spectrum relies primarily on nonlinear frequency down conversion from mature sources in the near infrared. Optical parametric oscillators and amplifiers (OPOs and OPAs) are among the favorite methods^10, 20, 21^, providing widely tunable, relatively broadband coverage (1000 cm^−1^) in both the shortwave infrared (SWIR), that is, the spectral range from 2000 to 5000 cm^−1^, and the longwave infrared (LWIR), from 500 to 2000 cm^−1^. In particular, simultaneous coverage of the 3–8 μm (−50 dB) range at over 400 mW average power has recently been achieved in an OPO^20^. However, these techniques often require the precise matching of the oscillators’ cavity lengths for OPOs or the delay arms in the OPA case, which increases complexity. Furthermore, the tuning of the central wavelength usually involves the mechanical rotation of the crystal and other optics, causing beam pointing issues. A significantly simplified scheme of frequency down conversion is intra-pulse difference frequency generation (DFG), where frequency conversion is achieved within a single beam, eliminating the need for interferometric temporal overlap. The low efficiency of this scheme, on the order of 0.01 to 1%, is offset by the potential for multi-octave-spanning spectrally coherent coverage while also providing intrinsic carrier-envelope phase stabilization^15^.

Until now, a mismatch between the transparency ranges of nonlinear crystals and the available laser sources has existed. Highly nonlinear non-oxide crystals, such as GaSe and AgGaSe, which transmit up to 25 μm and the low THz range^15^, are the most promising candidates for phase matching a broad bandwidth over a large fraction of their transparency windows. However, they should ideally be pumped at ~2 μm to achieve the desired broadband phase matching^22^ and to avoid parasitic two- or three-photon absorption. With standard ultrafast sources operating in the 1 μm wavelength range, this condition can only be met by using cascaded down-conversion schemes^23^, which tend to increase noise and compromise efficiency. To reach the LWIR using mature 1 μm sources as a direct pump, wide-bandgap crystals with relatively low nonlinearity^8^, such as LGS, are usually employed, leading to a two orders of magnitude lower figure of merit^23^ compared to direct pumping of GaSe at 2 μm, illustrating the compelling need for high power sources at 2 μm. Significant progress has recently been made with thulium fiber-based systems, with an output power of over 100 W and tens of micro-Joules generated at a MHz repetition rate^24^. Still, these systems require cascading stages of oscillator and amplifiers, often with free space stretcher and compressor units involved. An alternative approach based on Cr:ZnS/Se in a master-oscillator power-amplifier geometry has also demonstrated up to 1 MW peak powers directly from the oscillators and average output powers of ~7 W after amplification^4^.

In this work, we report the first mode-locked thin-disk oscillator operating in the 2 μm region that can provide high power output without further amplification. From the compact setup based on a Ho:YAG gain crystal, we obtained 18.7 Watt of average output power. This constitutes a tenfold increase over the power of any femtosecond oscillator demonstrated in the >1.5 μm region before^4, 16^, and the thin-disk concept promises further power scalability. To showcase the described advantages of the new 2 μm source, we demonstrate few-cycle pulse generation by means of soliton self-compression in an optical fiber^25^ and the subsequent LWIR generation by intra-pulse DFG, resulting in a two-octave-spanning spectrum (−30 dB) from 5 to 20 μm at an average power of 24 mW. Compared to our previous work^8^ based on mature Yb:YAG technology, where a measured average output power of 30 mW was obtained, a nearly twofold increase in bandwidth from 900 to 1750 cm^−1^ is demonstrated with our prototype source, illustrating the enormous potential of the 2 μm thin-disk approach to broadband MIR generation.

The setup of the thin-disk oscillator is shown in Figure 1. The Ho:YAG thin disk was pumped by a Tm-doped fiber laser at a wavelength of 1908 nm, resulting in a much smaller quantum defect and up-conversion process compared to the traditional Ho/Tm co-doped system. Rather than directly illuminating the thin disk, the pump light was first transmitted through a 550 μm-diameter multimode fiber to create a homogeneous pump spot and isolate the thulium fiber laser from the pump-head’s back reflection. The laser cavity had a four-pass configuration, which means that the laser beam passed through the disk eight times per round trip. This boost in round-trip gain enabled the use of higher output coupling ratios and increased the optical-to-optical efficiency. A Kerr medium and a hard aperture were used to enable Kerr-lens mode locking (KLM). With 3% output coupling, an average output power of 18.7 W was obtained under an estimated absorbed pump power of 83 W, which corresponds to an optical-to-optical efficiency of 22%. The average power demonstrated is currently limited only by the available pump power from a single Tm-fiber laser. The mode-locked spectrum and intensity autocorrelation trace are shown in Figure 2, exhibiting nearly Fourier transform-limited pulses with a pulse duration of 260 fs. The peak power, at approximately 0.8 MW, already approaches the 1 MW estimated for gain materials with much broader gain bandwidths such as Cr:ZnS^4^. The beat note signal shown in Figure 2c has a signal-to-noise ratio (SNR) of 78 dB, implying stable mode-locked operation of the oscillator, which was maintained for several hours on a day-to-day basis under ambient air conditions. The oscillator represents the first mode-locked thin-disk laser operating at wavelengths longer than 1.1 μm and delivers the highest average power of any mode-locked oscillator in the >1.5 μm range. It is also the first KLM oscillator based on the gain medium Ho:YAG and hence generates five times shorter pulses than previously reported Ho:YAG oscillators^26^.

The output from the oscillator, after passing through collimating lenses, was coupled into a commercial silica-core photonic-crystal fiber (PCF) for spectral broadening (Figure 3a). In contrast to the 1 μm wavelength, silica glass at 2 μm exhibits anomalous material dispersion, meaning that large-mode-area (LMA) PCFs with tens of micron core diameters can also be used for solitonic self-compression^27^, removing the need for broadband chirp mirrors while increasing the damage threshold. To obtain optimally compressed pulses, the input pulses’ peak power and the length and mode-field diameter of the fiber all need to match. This optimal combination was determined with the help of fiber simulations (see Methods). A 2.3 cm-long fiber with a core diameter of 12 μm was used at an incident power of 11 W, and 7 W was coupled out and collimated using a 90° off-axis parabolic gold mirror. The corresponding soliton order *N* was 6.7. The spectrum and pulse duration were characterized by an Fourier-transform Infrared (FTIR) spectrometer and a home-built SHG-FROG (see Methods). The spectrum extended from 1.7 to 2.5 μm (Figure 4a), and the retrieved pulse duration was 15 fs (Figure 4b), corresponding to 2.1 optical cycles at 2 μm. The symmetric FROG trace and the excellent retrieval indicate a highly reliable result. Similarly generated SHG pulses at 1 μm will also be attractive for future electro-optical sampling (EOS) of the generated LWIR spectrum. Although the fiber was not designed for light at 2 μm and was sustaining a peak power of 2 MW and an intensity of ~5 TW cm^−2^ near the output end, it did not show any damage after 4 weeks of daily continuous use at maximum power. In comparison, the same type of fiber was consistently damaged at approximately 1.55 TW cm^−2^ when irradiated with femtosecond pulses centered at 1.03 μm in another setup due to the higher photon energy and the increased probability of multi-photon absorption, which initiates fiber damage.

A 1 mm-thick GaSe crystal was used to generate MIR radiation via intra-pulse DFG. GaSe was chosen due to its large nonlinear coefficient (*d*_22_=54 pm/V at 10.6 μm) and birefringence (*B*=0.375 at 10.6 μm)^28^. It is transparent in a broad wavelength range from 0.65 to 20 μm and even up to 30 μm for shorter crystals^29^. In addition, it has a high optical damage threshold^22^, high thermal conductivity and minimal two-photon absorption at a pump wavelength of ~0.6 eV and is readily available commercially. For these reasons, it has been frequently used for MIR and THz generation and detection via EOS^10, 29^. Additionally, in contrast to the LGS crystals employed in^8^, GaSe crystals are available from multiple suppliers in reproducible quality.

The experimental setup for MIR generation is depicted in Figure 3b. The self-compressed pulses were focused into the GaSe crystal with a spot diameter of 94 μm. Due to the high reflection loss of the uncoated GaSe, only 5.6 W of light entered the crystal, corresponding to a peak intensity of 45 GW cm^−2^, assuming a Gaussian spatial beam profile and taking into account the pedestal in the temporal profile. Type I phase matching, which has a broader matching bandwidth than Type II phase matching in GaSe^22^, was fulfilled by rotating the crystal and the half-wave plate such that the incident polarized beam was projected equally onto the extraordinary and ordinary crystal axes. The generated LWIR radiation was separated from the transmitted driving beam by a long-pass filter and sent to diagnostics. The output spectrum, which could be optimized by tuning the incident angle between the beam and the crystal, was measured using a monochromator (see Methods). As shown in Figure 5a, the spectrum spans two octaves and covers the wavelength range from 5 to 20 μm (−30 dB)—a bandwidth that can support few-cycle pulses in the LWIR. It should be noted that the spectrum at longer wavelengths (starting from 11 μm) was strongly affected by the low transmission of the ZnSe lens and long-pass filter and the low dispersion efficiency of the monochromator grating. Moreover, according to our one-dimensional numerical beam propagation, the spectrum is likely to extend toward 30 μm. The characterization and separation of the entire 4–30 μm infrared range from the 2 μm driving radiation are challenging, but further investigation in this direction is being planned. An average power of 24 mW was measured (S302C, Thorlabs; see Supplementary Information) behind a long-pass filter with a cut-on wavelength of 4.5 μm. Considering that the filter has a transmission of <90% above 4.5 μm, we conclude that the average power directly after the crystal was over 27 mW. At this power level, the entire multi-octave spanning spectrum will be useful for spectroscopic applications, especially when combined with field-resolved techniques that provide a significantly higher dynamic range than traditional methods^29, 30^. The generated LWIR inside the crystal is estimated to be 34 mW considering the reflection loss of the GaSe crystal. The MIR beam has excellent spatial quality (Figure 5 inset), with brightness levels exceeding those in large-scale synchrotron facilities^8^. With all components included, the current setup has a footprint of 1.8 × 0.6 m and can be reduced further.

In conclusion, we have developed a novel KLM Ho:YAG thin-disk oscillator capable of generating 18.7 W of average power. This is the highest power ever reported, by one order of magnitude, for a mode-locked oscillator at wavelengths longer than 2 μm. The output was subsequently coupled into a silica LMA-PCF to induce soliton self-compression. The compressed pulse duration was measured to be 15 fs—equivalent to 2.1 optical cycles. This output was focused into a GaSe crystal where intra-pulse DFG occurred, providing a continuous spectrum spanning from 4.5 to 20 μm at over 24 mW of average power. The inherently CEP stable, two-octave spanning spectrum already covers the entire molecular fingerprint region (500–1500 cm^−1^) and a significant portion of the functional group region (1500–4000 cm^−1^). A vast array of spectroscopic techniques, including time-resolved pump-probe spectroscopy, s-SNOM nanoscopy and, potentially, dual comb spectroscopy, will benefit from the improved coverage and increased power from our compact source. The thin-disk geometry of the Ho:YAG crystal is inherently power scalable, and improvement in power well beyond that provided by this first demonstration is expected. Further optimization of the fiber self-compression stage by reducing the fiber core diameter should also result in an even broader seed spectrum, possibly extending the MIR spectrum’s high frequency edge to above 3000 cm^−1^, where different polymers and lipids have strong absorption lines. Together with the good power handling capability of GaSe at a 2 μm pump, we expect the MIR output, in terms of both power and spectral reach, to be significantly improved in the future. The reported development thus opens the way to a compact, robust and ultra-broadband MIR frequency comb source, benefiting existing and emerging approaches in spectroscopy^30^.

## Methods

### Kerr-lens mode-locked Ho:YAG thin-disk oscillator

**Figure 1:**
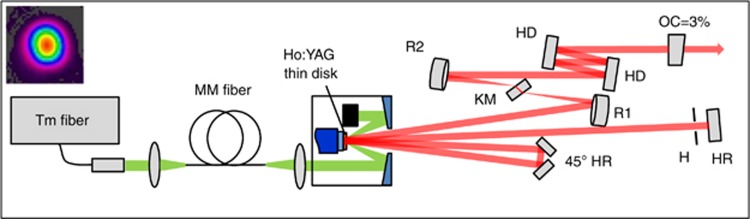
Mode-locked Ho:YAG thin-disk oscillator. HR, high-reflection mirrors; R1 and R2, concave spherical mirrors; KM, Kerr medium; HD, high dispersion mirrors; H, hard aperture; OC, output coupler. The oscillator contains a wedged Ho:YAG thin disk with a thickness of ~200 μm and 2.5 at.% Ho doping, housed within a multi-pass pump head (TRUMPF Laser GmbH). The pump spot diameter on the thin disk is 2.5 mm. The thin disk is placed as a folding mirror in a cavity based on a Z-shaped design. A pair of 45° high-reflection mirrors folds the beam path to provide eight passes through the thin disk per round trip. A 1 mm-thick sapphire plate acting as the Kerr medium is placed at the focus between two concave mirrors (R1 and R2). The total anomalous group delay dispersion per round trip is −16000 fs^2^, introduced by a pair of chirped mirrors with up to 4 bounces on each surface. A water-cooled copper plate with a circular hole is placed near an end mirror as a hard aperture to aid the stabilization of KLM. The total cavity length is ~1960 mm, corresponding to a repetition rate of 77 MHz. Inset: the beam profile of the oscillator output.

Before initiating mode locking, the oscillator was operated in the continuous-wave (CW) regime. With a 3% output coupler, an average output power of 17 W was reached at an estimated absorbed pump power of ~83 W. This was obtained close to the cavity stability edge, where KLM could be initiated even though the oscillator provided a higher output power of 20 W when operated close to the center of the stability zone (Supplementary Fig. 1). The stability edge was approached by increasing the distance between mirrors R1 and R2 (Figure 1). Mode locking was initiated by perturbing a translation stage on which the output coupler mirror was mounted. Once mode-locked, the average output power was increased to 18.7 W, corresponding to an optical-to-optical efficiency of 22%.

### FROG measurement of the compressed pulses

**Figure 4:**
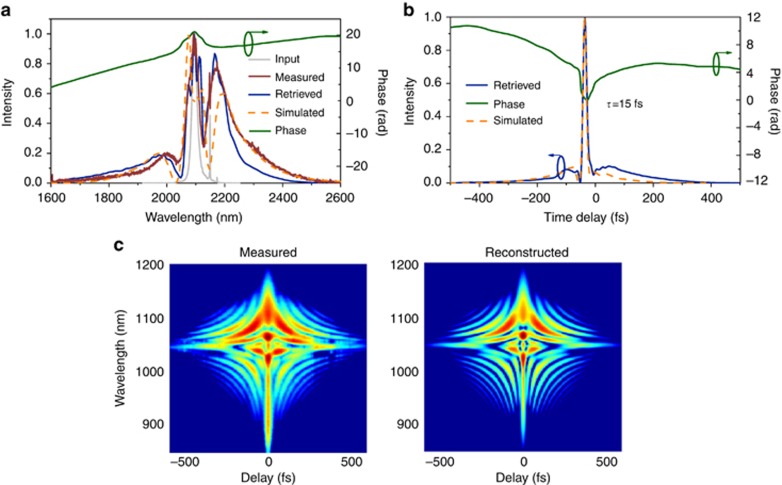
Characterization of the self-compressed driving pulses. (**a**) The input, measured, and retrieved spectra of the self-compressed driving pulse and the simulated spectrum (see Methods). The spectra extend from 1700 to 2500 nm. (**b**) The retrieved temporal intensity and phase measured by a home-built SHG-FROG, and the simulated pulse duration. The retrieved FWHM pulse duration of 15 fs agrees well with the Fourier transform limit of 15.5 fs, which is within measurement uncertainty. (**c**) The measured and retrieved SHG-FROG traces.

The pulse duration of 15 fs was measured using a home-built apparatus based on second-harmonic frequency-resolved optical gating (FROG). The incident beam was separated into two parts by a D-shaped silver mirror. The separated beams were then non-collinearly focused into a 50 μm-thick BBO crystal by a 90° off-axis parabolic mirror (effective focal length=2″). Upon optimized spatial overlap, a delay-dependent FROG signal was generated by scanning one arm length with a piezo stage. The generated signal was isolated from the two original beams and focused into a multimode fiber feeding into an optical spectrometer with a resolution of 0.5 nm (Ocean Optics, HR4000). To correct for systematic errors, the fundamental spectrum at the BBO crystal position was measured using an FTIR spectrometer and used as the marginal input in the retrieval algorithm. Figure 4a shows the comparison of the measured and the retrieved FROG spectra, indicating a reliable retrieval. The FROG error of the retrieved pulse was 0.8% with a 512 × 512 grid size.

### MIR spectrum measurement

The MIR beam was focused into a Czerny-Turner ¼ m monochromator by an AR-coated ZnSe lens with a focal length of 100 mm. A mechanical chopper and a lock-in amplifier were used together with the monochromator to modulate the intensity of the light beam and improve the signal-to-noise ratio. The grating inside the monochromator, with 75 grooves per mm, has a blaze wavelength of 7 μm and provides a tuning range from 4.5 to 20 μm. Its reciprocal dispersion is 51.7 nm mm^−1^, which corresponds to a resolution of 85 nm because the entrance and exit slits were set to a width of 1.7 mm. To suppress signals from second-order diffractions, three long-pass filters with cut-on wavelengths at 4.5, 7.3 and 11 μm were used to record three different spectra spanning from 4.5 to 8.5 μm, from 6 to 13 μm and from 10 to 22 μm. These raw spectra were then stitched together.

### Simulation of pulse propagation in fiber

The fiber simulation was conducted using a commercially available software (FiberDesk), which solves the generalized nonlinear Schrödinger equation^25^, including the Raman response and the self-steepening effect. The dispersion of the fiber was set at −0.123 ps^2^ m^−1^ at 2100 nm, with no higher-order dispersions included. The nonlinear refractive index *n*_*2*_ was 2.2 × 10^−20^ m^−2^ W^−1^^ref. 25^. The Raman fraction fR was set at 0.18, with the Raman response parameters τ_1_ and τ_2_ being 12.2 and 32 fs, respectively^25^. The mode-field diameter was assumed to be 10.8 μm at 2100 nm. A Gaussian pulse with a FWHM duration of 260 fs was used as input with a pulse energy of 0.91 μJ.

## Author contributions

The main setup was designed and built by JZ, KM and OP. The measurements were taken by JZ, KM and NN. The self-compression experiment was designed and realized by JZ, MS and KM. The customized thin-disk modules were prepared by DB and DS. The specialized dielectric optics were designed and fabricated by VP. The experiment was conceived by FK and OP. The project was coordinated by OP. All authors reviewed and contributed to the final manuscript.

## Figures and Tables

**Figure 2:**
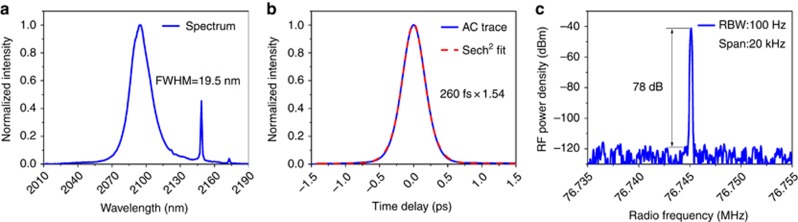
Pulse characterization. (**a**) Mode-locked spectrum measured with a Fourier transform infrared (FTIR) spectrometer (Bristol 721), showing a full-width at half-maximum (FWHM) spectrum bandwidth of 19.5 nm. The bandwidth slightly exceeds the fluorescence spectrum of the Ho:YAG crystal at FWHM. (**b**) Intensity autocorrelation trace. The pulse duration is 260 fs assuming a sech^2^ shape autocorrelation fit, indicating nearly Fourier transform-limited pulses (time-bandwidth product: 0.34). (**c**) Radio frequency spectrum measured using a radio frequency analyzer with a resolution bandwidth of 100 Hz. It shows a high extinction of ~78 dB, implying stable mode-locked operation.

**Figure 3:**
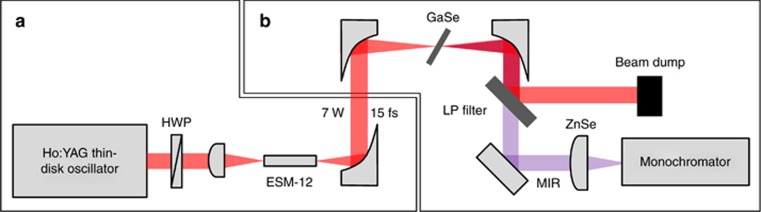
Self-compression and MIR generation setup. (**a**) Self-compression stage, providing driving pulses for MIR generation. The pulses from the oscillator are coupled into a commercial silica-core photonic-crystal fiber by an aspherical lens with a focal length of 8 mm. The fiber is 2.3 cm long with a core diameter of 12 μm (ESM-12, NKT). The output from the fiber is collimated using a gold 90° off-axis parabolic (OAP) mirror with an effective focal length (EFL) of 25 mm. A half-wave plate (HWP) is placed in front of the coupling lens to change the polarization of the driving pulses. (**b**) MIR generation setup. The driving pulses are subsequently focused into the GaSe crystal by another gold 90° OAP mirror with an EFL of 20 mm. The generated MIR radiation propagates collinearly with the driving beam transmitted through the crystal. Both beams are collimated by another gold OAP mirror with an EFL of 33 mm and separated by a long-pass filter. The MIR beam is then sent to the monochromator for spectral characterization (see Methods).

**Figure 5:**
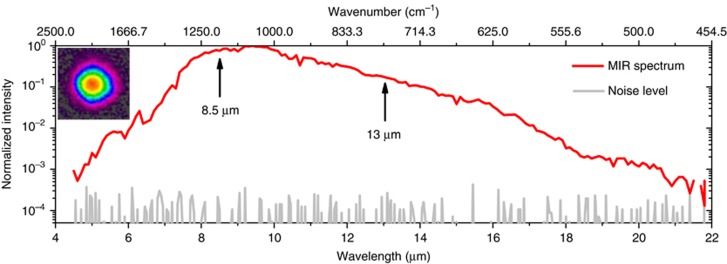
MIR spectrum and beam profile. The MIR spectrum (red line) together with the noise floor (gray line) measured using a monochromator. The MIR spectrum extends from 500 cm^−1^ to 2250 cm^−1^ (−30 dB), corresponding to the wavelength range from 4.5 to 20 μm. Inset: the beam profile measured using a Pyrocam beam profiler.

## References

[bib1] Eisele M, Cocker TL, Huber MA, Plankl M, Viti L et al. Ultrafast multi-terahertz nano-spectroscopy with sub-cycle temporal resolution. Nat Photon 2014; 8: 841–845.

[bib2] Amenabar I, Poly S, Nuansing W, Hubrich EH, Govyadinov AA et al. Structural analysis and mapping of individual protein complexes by infrared nanospectroscopy. Nat Commun 2013; 4: 2890. 24301518 10.1038/ncomms3890 PMC3863900

[bib3] Bechtel HA, Muller EA, Olmon RL, Martin MC, Raschke MB. Ultrabroadband infrared nanospectroscopic imaging. Proc Natl Acad Sci USA 2014; 111: 7191–7196. 24803431 10.1073/pnas.1400502111 PMC4034206

[bib4] Vasilyev S, Moskalev I, Mirov M, Smolski V, Mirov S et al. Ultrafast middle-IR lasers and amplifiers based on polycrystalline Cr:ZnS and Cr:ZnSe. Opt Mater Express 2017; 7: 2636–2650.

[bib5] Dudley JM, Taylor JR. Supercontinuum Generation in Optical Fibers. Cambridge: Cambridge University Press; 2010.

[bib6] Holzwarth R, Udem T, Hänsch TW, Knight JC, Wadsworth WJ et al. Optical frequency synthesizer for precision spectroscopy. Phys Rev Lett 2000; 85: 2264–2267. 10977987 10.1103/PhysRevLett.85.2264

[bib7] Petersen CR, Møller U, Kubat I, Zhou BB, Dupont S et al. Mid-infrared supercontinuum covering the 1.4–13.3 μm molecular fingerprint region using ultra-high NA chalcogenide step-index fibre. Nat Photon 2014; 8: 830–834.

[bib8] Pupeza I, Sánchez D, Zhang J, Lilienfein N, Seidel M et al. High-power sub-two-cycle mid-infrared pulses at 100 MHz repetition rate. Nat Photon 2015; 9: 721–724.

[bib9] Schliesser A, Picqué N, Hänsch TW. Mid-infrared frequency combs. Nat Photon 2012; 6: 440–449.

[bib10] Keilmann F, Amarie S. Mid-infrared frequency comb spanning an octave based on an Er Fiber Laser and difference-frequency generation. J Infrared Milli Terahz Waves 2012; 33: 479–484.

[bib11] Haas J, Mizaikoff B. Advances in mid-infrared spectroscopy for chemical analysis. Ann Rev Anal Chem 2016; 9: 45–68. 10.1146/annurev-anchem-071015-041507 27070183

[bib12] Shirai H, Yeh TT, Nomura Y, Luo CW, Fuji T. Ultrabroadband midinfrared pump-probe spectroscopy using chirped-pulse up-conversion in gases. Phys Rev Appl 2015; 3: 051002.

[bib13] Bakker HJ, Skinner JL. Vibrational spectroscopy as a probe of structure and dynamics in liquid water. Chem Rev 2010; 110: 1498–1517. 19916491 10.1021/cr9001879

[bib14] Dantus M, Lozovoy VV. Experimental coherent laser control of physicochemical processes. Chem Rev 2004; 104: 1813–1860. 15080713 10.1021/cr020668r

[bib15] Huber R, Brodschelm A, Tauser F, Leitenstorfer A. Generation and field-resolved detection of femtosecond electromagnetic pulses tunable up to 41 THz. Appl Phys Lett 2000; 76: 3191–3193.

[bib16] Mirov SB, Fedorov VV, Martyshkin D, Moskalev IS, Mirov M et al. Progress in mid-IR lasers based on Cr and Fe-doped II-VI chalcogenides. IEEE J Sel Top Quantum Electron 2015; 21: 292–310.

[bib17] Rauter P, Capasso F. Multi-wavelength quantum cascade laser arrays. Laser Photon Rev 2015; 9: 452–477.

[bib18] Ellis GJ, Martin MC. Opportunities and challenges for polymer science using synchrotron-based infrared spectroscopy. Eur Polym J 2016; 81: 505–531.

[bib19] Nomura Y, Shirai H, Ishii K, Tsurumachi N, Voronin AA et al. Phase-stable sub-cycle mid-infrared conical emission from filamentation in gases. Opt Express 2012; 20: 24741–24747. 23187238 10.1364/OE.20.024741

[bib20] Smolski V, Vasilyev S, Moskalev I, Mirov M, Muraviev A et al Sub-watt femtosecond laser source with the spectrum spanning 3–8 μm. in Laser Congress 2017 (ASSL, LAC), OSA Technical Digest (online) (Optical Society of America, 2017), paper AM4A.6.

[bib21] Steinle T, Mörz F, Steinmann A, Giessen H. Ultra-stable high average power femtosecond laser system tunable from 1.33 to 20 μm. Opt Lett 2016; 41: 4863–4866. 27805636 10.1364/OL.41.004863

[bib22] Sell A. Nichtlineare spektroskopie mit einer hochintensiven THz-lichtquelle: wechselwirkungen mit ladungsträgern und spins PhD thesis, University of Konstanz, München, Germany, 2010.

[bib23] Petrov V. Parametric down-conversion devices: The coverage of the mid-infrared spectral range by solid-state laser sources. Opt Mater 2012; 34: 536–554.

[bib24] Gaida C, Gebhardt M, Stutzki F, Jauregui C, Limpert J et al. Thulium-doped fiber chirped-pulse amplification system with 2 GW of peak power. Opt Lett 2016; 41: 4130–4133. 27607990 10.1364/OL.41.004130

[bib25] Agrawal GPOptical solitons In: Agrawal GPNonlinear Fiber Optics. 4th ed.San Diego: Academic Press; 2006, pp 120–pp176.

[bib26] Wang YC, Lan RJ, Mateos X, Li J, Hu C et al. Broadly tunable mode-locked Ho:YAG ceramic laser around 2.1 μm. Opt Express 2016; 24: 18003–18012. 27505767 10.1364/OE.24.018003

[bib27] Gaida C, Gebhardt M, Stutzki F, Jauregui C, Limpert J et al. Self-compression in a solid fiber to 24 MW peak power with few-cycle pulses at 2 μm wavelength. Opt Lett 2015; 40: 5160–5163. 26565824 10.1364/OL.40.005160

[bib28] Vodopyanov KL. Parametric generation of tunable infrared radiation in ZnGeP_2_ and GaSe pumped at 3 μm. J Opt Soc Am B 1993; 10: 1723–1729.

[bib29] Kübler C, Huber R, Tübel S, Leitenstorfer A. Ultrabroadband detection of multi-terahertz field transients with GaSe electro-optic sensors: Approaching the near infrared. Appl Phys Lett 2004; 85: 3360–3362.

[bib30] Pupeza I, Schweinberger W, Trubetskov M, Hussain SA, Vamos L et al Background-free sensing of molecular vibrations by field-resolved spectroscopy. in 6th International Conference onAttosecond Physics. 2–7 July 2017;Xi'an, China.

