# Supplementary material for: Multi-mW, few-cycle mid-infrared continuum spanning from 500 to 2250 cm−1
Source: Light Sci Appl. 2018 Feb 23;7:17180–. doi: 10.1038/lsa.2017.180 (PMC6060060; doi:10.1038/lsa.2017.180)
Supplement: Supplementary Information [file lsa2017180x1.docx]

**Multi-mW, few-cycle mid-infrared continuum spanning from 500 to 2250 cm^-1^**

**Supplementary**

**Supplementary figure 1 | Average CW output power vs. estimated absorbed pump power.** The oscillator was running close to the center of the stability zone. Average output power of 20 W is obtained under an estimated pump power of 83 W.

**Power measurement and calibration of the MIR beam**

The sensor used for the average power measurements is S302C (Thorlabs), which is specified for 100 μW-2 W power and 0.19-25 μm spectral range. Its measurement uncertainty is ±5%@190-2940 nm. The sensor has been purchased in July 2017 and, thus, is freshly calibrated. We have also measured the average output power with a sensor from Coherent (PowerMax PS19). The measured values coincide within 5% uncertainty value.
